# Oncolytic bacteria therapy for malignant glioma

**DOI:** 10.3389/fimmu.2026.1951763

**Published:** 2026-09-08

**Authors:** Feiyue Li, Jing Zhao, Ang Ji, Junlong Zhao, Sanzhong Li

**Affiliations:** 1 Department of Neurosurgery, Xijing Hospital, Fourth Military Medical University, Xi’an, China; 2 School of Basic Medical Sciences, Shanxi Medical University, Taiyuan, China; 3 Department of Anesthesia, Xijing Hospital, Fourth Military Medical University, Xi’an, China; 4 State Key Laboratory of Holistic Integrative Management of Gastrointestinal Cancers, Department of Medical Genetics and Developmental Biology, Fourth Military Medical University, Xi’an, China

**Keywords:** genetic engineering, glioma, immunotherapy, oncolytic bacteria, synthetic biology, tumor microenvironment

## Abstract

Malignant glioma is a group of primary malignant tumors of the central nervous system with an extremely poor prognosis, with glioblastoma being the most malignant type. Its highly invasive nature, tumor heterogeneity, stubborn immunosuppressive microenvironment, and the barrier effect of the blood-brain barrier result in limited efficacy of traditional surgery, radiotherapy, and chemotherapy, leading to high recurrence rates and short survival times. The tumor hypoxic microenvironment, as a core cause of treatment resistance and recurrence in glioma, also provides a natural therapeutic target for novel targeted therapies. Oncolytic bacteria, as an emerging tumor biotherapy technology, leverage natural tumor hypoxia targeting, autonomous proliferation capacity, multiple antitumor mechanisms, and flexible genetic modification potential. They can specifically colonize deep hypoxic areas of glioma, directly lyse tumor cells, remodel the tumor immune microenvironment, and overcome the bottleneck of drug delivery across the blood-brain barrier, making them a research hotspot for overcoming the challenges of glioma treatment. This article systematically reviews the core strain characteristics, antitumor molecular mechanisms, genetic engineering and synthetic biology modification strategies of oncolytic bacteria, summarizes preclinical research results and early clinical trial progress, deeply analyzes the core challenges currently faced in clinical translation, such as biosafety, delivery efficiency, and efficacy evaluation, and looks forward to future development directions combined with multidisciplinary cross-technologies, aiming to provide comprehensive theoretical support and practical references for deepening basic research and clinical translation of oncolytic bacteria in the field of malignant glioma.

## 1 Introduction

Malignant glioma is the most common intracranial primary malignant tumor in adults, with glioblastoma (GBM) having the highest degree of malignancy. Clinical data show that the median survival time of patients is only 14–15 months, and the 5-year survival rate is extremely low, making it a stubborn disease urgently needing to be overcome in the field of neurosurgery (1). The current standard clinical treatment is maximal safe surgical resection combined with postoperative concurrent chemoradiotherapy. However, this system has significant treatment bottlenecks: gliomas grow diffusely infiltratively with blurred boundaries from normal brain tissue, making it difficult to completely remove the lesion surgically; high tumor heterogeneity and the presence of glioma stem cells lead to frequent chemoradiotherapy resistance; the physical barrier of the blood-brain barrier (BBB) greatly reduces the efficiency of drug delivery into the brain; and the tumor microenvironment exhibits a highly immunosuppressive state, forming a “cold tumor” characteristic, which greatly limits the efficacy of traditional immunotherapy (2).

**Figure 1:**
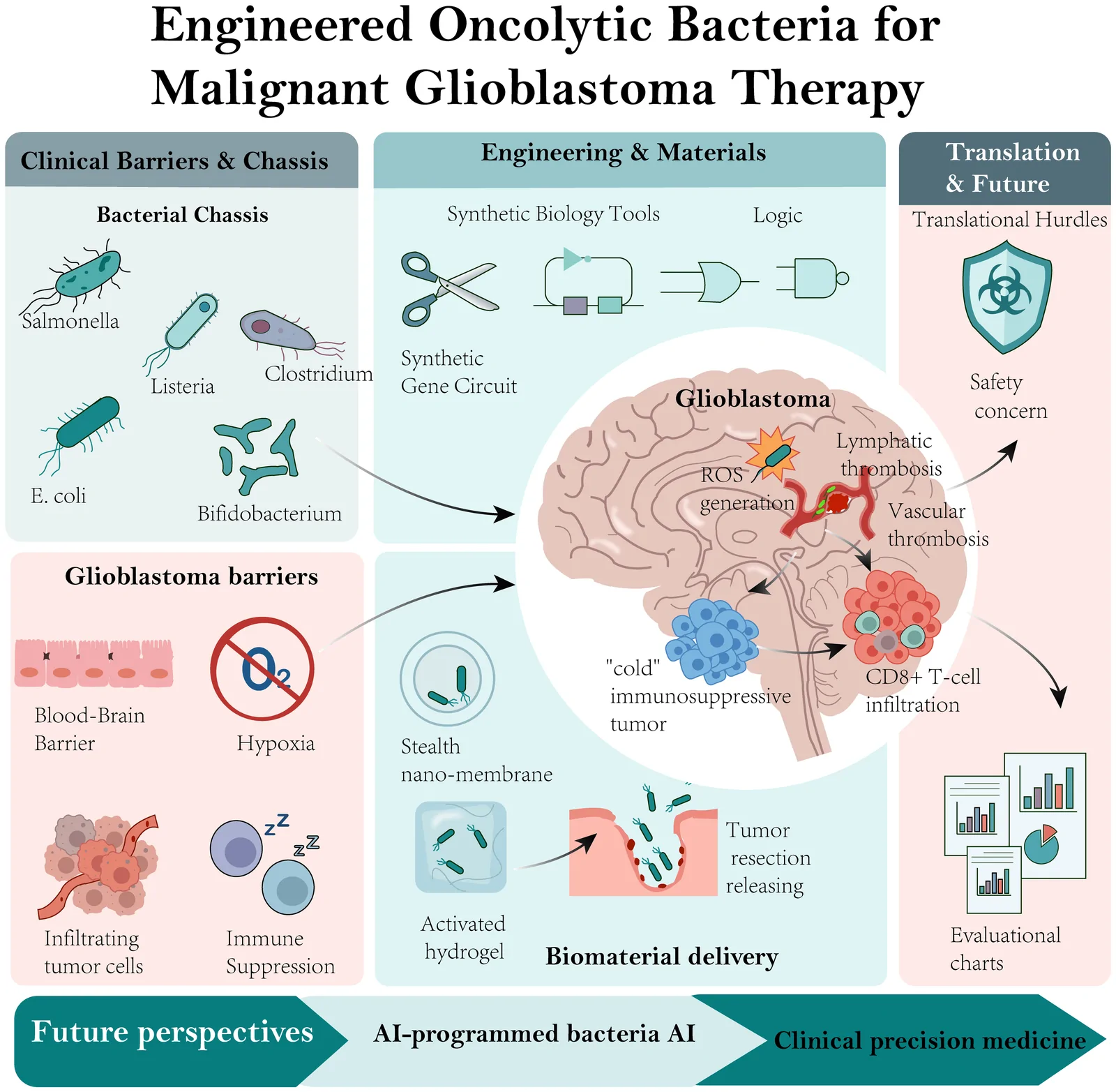
Integrated research framework of engineered oncolytic bacteria for glioblastoma therapy. This schematic presents a full ecosystem from strain engineering to clinical translation, including five interrelated sections: (1) Left panel: Bacterial chassis and intrinsic glioma therapeutic barriers; (2) Central area: Synthetic biology tools, genetic circuits and biomaterial delivery platforms; (3) Tumor core: Multiple anti-tumor mechanisms of oncolytic bacteria; (4) Right panel: Clinical translational hurdles and evaluation systems; (5) Bottom axis: Future directions of AI-programmable bacteria and precision immunotherapy.

In recent years, tumor immunotherapy has developed rapidly, and oncolytic virus therapy has achieved encouraging preclinical and early clinical progress in glioma treatment. Meanwhile, oncolytic bacteria therapy has achieved technological innovation and research revival due to its unique advantages. Compared with oncolytic viruses, oncolytic bacteria have a larger genome capacity, simpler genetic manipulation, autonomous proliferation without relying on tumor cell surface receptor infection, and can precisely target the characteristic hypoxic microenvironment of gliomas, possessing multiple functions including direct oncolysis, immune activation, and targeted drug delivery (3). With the iterative upgrading of synthetic biology and genetic engineering technologies, the virulence defects of wild-type bacteria have been effectively improved, and the targeting, safety, and therapeutic efficacy of engineered oncolytic bacteria have been significantly enhanced, showing excellent tumor suppression and prognosis improvement effects in preclinical glioma models. Based on the latest research results, this article comprehensively reviews the biological mechanisms, strain application characteristics, engineering modification strategies, and clinical research progress of oncolytic bacteria in treating malignant glioma, systematically analyzes existing translational barriers, and proposes targeted solutions, providing a reference for the clinical implementation and optimization of this technology. The complete research system covering bacterial chassis, biological delivery and translational challenges for oncolytic bacterial glioblastoma therapy is summarized (**Figure 1**).

## 2 Treatment dilemmas of malignant glioma and the intervention logic of oncolytic bacteria

### 2.1 Core bottlenecks of traditional treatment

The refractory nature of glioma stems from its unique biological characteristics and the multiple barriers of the lesion microenvironment. First, tumor heterogeneity and invasive growth: Glioma cells exhibit significant genetic and epigenetic differences. Glioma stem cells possess self-renewal and multi-directional differentiation capabilities, which are the core sources of treatment resistance and recurrence. Moreover, tumor cells diffusely infiltrate normal brain tissue, making complete surgical resection extremely difficult (4). Second, the barrier effect of the blood-brain barrier: The intact BBB strictly limits the entry of macromolecular drugs and most small-molecule chemotherapeutic drugs into the brain parenchyma. Even though tumor growth increases the permeability of the blood-tumor barrier, it still cannot meet the required effective therapeutic drug concentration (5). Third, the immunosuppressive microenvironment: The glioma microenvironment is enriched with immunosuppressive cells such as regulatory T cells and M2-type tumor-associated macrophages, lacking effector T cell infiltration, forming an immune-privileged state, which significantly reduces the efficacy of mainstream immunotherapies like immune checkpoint inhibitors (6). Fourth, hypoxia-mediated treatment resistance: The internal vascular development of glioma is disordered, with insufficient blood perfusion, forming large areas of hypoxic necrosis. The hypoxic environment activates signaling pathways such as HIF-1α, inducing epithelial-mesenchymal transition and chemoradiotherapy resistance in tumor cells, creating a “tumor sanctuary” inaccessible to traditional treatments (7).

Notably, among the therapeutic bottlenecks enumerated above, the blood–brain barrier and the immune-privileged state of the central nervous system represent core features that distinguish GBM from peripheral solid tumors. Although the hypoxic microenvironment and tumor heterogeneity are shared by GBM and extracranial malignancies, they are more severe and dynamically variable in GBM: the ubiquitous overexpression of HIF-1α renders the hypoxic landscape substantially more intense, while the tumor heterogeneity exhibits more dramatic spatiotemporal fluctuations.

### 2.2 Natural advantages of oncolytic bacteria therapy

The biological characteristics of oncolytic bacteria are highly compatible with the pathological features of glioma, and may address several limitations of conventional therapies and possessing unique therapeutic advantages. First, hypoxia targeting specificity: Facultative and obligate anaerobic bacteria can specifically sense the tumor hypoxic microenvironment and colonize the core necrotic area of glioma, while they cannot survive or are quickly cleared in the oxygen-rich environment of normal brain tissue, exhibiting natural tumor targeting precision (8). Second, autonomous proliferation and long-term tumor suppression: As “living drugs,” bacteria can autonomously proliferate within the tumor, dynamically adapting to the tumor growth rhythm, continuously maintaining local therapeutic concentrations, overcoming the shortcomings of traditional drugs with short half-lives requiring repeated administration (9). Third, multiple antitumor mechanisms: They can directly kill tumor cells through nutrient competition and cell lysis, while indirectly inhibiting tumors by activating innate and adaptive immunity, destroying tumor blood vessels, and remodeling the microenvironment, suppressing tumor proliferation and recurrence from multiple dimensions (10).

Fourth, efficient gene delivery vector: Bacteria have a strong genome carrying capacity and can be loaded with therapeutic genes and immunomodulatory genes through gene editing, achieving precise *in-situ* “drug manufacturing” within the tumor, overcoming the drug delivery problem of the BBB (11). Fifth, controllable safety: After genetic attenuation modification, the pathogenicity of bacteria is greatly reduced, and they exhibit relatively preferential enrichment in tumor tissues, with no obvious damage to normal brain tissue observed under the reported animal models and administration conditions, and adverse reactions are manageable (12).

## 3 Characteristics of mainstream oncolytic bacterial strains and their application in glioma treatment

Based on oxygen tolerance characteristics, oncolytic bacteria are mainly divided into two categories: facultative anaerobes and obligate anaerobes. The colonization characteristics, oncolytic mechanisms, and safety profiles of these two types of strains differ significantly, forming a differentiated application pattern in glioma treatment. Meanwhile, some probiotic strains also show unique adjuvant therapeutic potential.

### 3.1 Facultative anaerobes-attenuated salmonella

Attenuated *Salmonella* Typhimurium is currently the most extensively studied and widely used facultative anaerobic oncolytic strain. It can colonize both the hypoxic core and the oxygen-rich peripheral proliferative zone of glioma, possessing broad-spectrum antitumor activity and convenient genetic modification advantages (13). The classic strain VNP20009, by knocking out the *msbB* and *purI* virulence and metabolic genes, significantly reduces endotoxin toxicity and the risk of systemic infection while retaining excellent tumor-targeting colonization ability, making it a model strain for glioma research (14).

In glioma treatment, *Salmonella* can act through multiple pathways: depriving tumor cells of core nutrients like glucose and amino acids through nutrient competition, inhibiting tumor proliferation; secreting cytotoxins to activate the caspase-3 apoptotic pathway in tumor cells, inducing apoptosis; and downregulating P-glycoprotein expression in tumor cells, reversing temozolomide chemotherapy resistance (15);Attenuated *Salmonella* can respond to metabolites and physicochemical signals in the tumor microenvironment. However, the contribution of classical chemotaxis and motility to tumor colonization remains controversial. In mouse models, Stritzker et al. reported that colonization of *Escherichia coli* within tumors depends primarily on bacterial metabolism and interactions with macrophages, whereas chemotaxis and motility are not essential for efficient bacterial accumulation in tumors. Thus, although chemotaxis is considered a potential mechanism for bacterial tumor targeting, quantitative studies indicate that its contribution to tumor colonization is limited. Other factors, such as passive enrichment via vascular leakage and preferential proliferation within the tumor microenvironment, likely play a more dominant role (16). Preclinical orthotopic glioma models have confirmed that engineered *Salmonella*, after intravenous administration, specifically enriches in brain tumor tissue, significantly reducing tumor volume and prolonging the survival of tumor-bearing animals, with minimal bacterial colonization in normal brain tissue, demonstrating excellent targeting safety (17). The preclinical data outlined above highlight the unique advantages of *Salmonella* in GBM therapy. Unlike peripheral solid tumors such as hepatocellular carcinoma and melanoma, the foremost prerequisite for therapeutic success in glioma lies in whether the drug can cross the blood–brain barrier. The intrinsic barrier-crossing capability of *Salmonella* enables it to actively reach intracranial tumor lesions-a trait that, while not essential for peripheral tumors, directly determines the feasibility of GBM treatment. At the immune activation level, *Salmonella* can induce pyroptosis in glioma cells, releasing tumor antigens and damage-associated molecular patterns, thereby activating innate and adaptive antitumor immune responses (18). Engineered *Salmonella* can also utilize the lag effect of IL-10 receptor on tumor-infiltrating immune cells-where a transient increase in IL-10 within the tumor microenvironment causes CD8^+^ T cells, tumor-associated macrophages, and neutrophils to maintain high IL-10R expression for a prolonged period. After *Salmonella* colonization, it stimulates macrophages to secrete IL-10, which on one hand expands and activates tumor-resident CD8^+^ T cells, and on the other hand inhibits the phagocytic clearance by neutrophils, achieving the dual goal of “escaping innate immune clearance while activating antitumor immunity” (19).

However, the metabolic regulatory effect of *Salmonella* has a “double-edged sword” effect. *Salmonella* depletes asparagine in the microenvironment via asparaginase, which can inhibit the c-Myc pathway in tumor cells, thereby weakening tumor metabolic activity. However, it also destabilizes the c-Myc protein in infiltrating T cells, causing metabolic reprogramming blockade and functional inhibition in T cells-this discovery reveals the inherent trade-off between the “tumor-suppressive effect and T cell immune sacrifice” of *Salmonella* (20). Therefore, this strain has certain limitations: excessive attenuation of strain virulence will simultaneously weaken intratumoral proliferation and oncolytic ability. For immunocompromised glioma patients, precise gene editing is still needed to further optimize the safety window (21). To overcome the clinical limitations of VNP20009, researchers constructed a new ppGpp synthesis-deficient strain HCS1 by knocking out the relA and spoT genes in VNP20009. Its median lethal dose is approximately 72 times higher than that of VNP20009, allowing safer administration at higher doses, and it demonstrates stronger tumor suppression and immune activation effects in various solid tumor models (22). This strategy provides a new direction for further optimizing *Salmonella* strains for glioma treatment.

### 3.2 Obligate anaerobes – Clostridium

*Clostridium* is a strict obligate anaerobe that can only survive and proliferate in anoxic or very low oxygen environments. It is an ideal strain for targeting the deep hypoxic necrotic area of glioma. The core representative is the attenuated *Clostridium novyi* strain C. *Clostridium novyi*-NT, which, by knocking out the major lethal alpha-toxin gene, retains hypoxic proliferation ability while greatly reducing pathogenicity (23). This strain is administered in spore form, can tolerate the systemic blood circulation environment, and germinates and proliferates massively predominantly in the hypoxic core of glioma. It destroys host cell membrane integrity and lyses tumor cells through physical cell rupture and secretion of metabolites like phospholipase C and lipolytic enzymes, progressively clearing drug-resistant tumor tissue from the center outward, precisely targeting the hypoxic resistant areas inaccessible to traditional chemoradiotherapy (24, 25).

Simultaneously, the proliferation of *C. novyi*-NT can induce local inflammation, recruiting a large number of immune cells such as neutrophils to the tumor periphery. While limiting bacterial spread, this also triggers an antitumor immune response, helping to clear residual tumor cells at the tumor margin (26). Furthermore, it disrupts the abnormal tumor vascular structure, forming local thrombi, cutting off the tumor’s nutrient supply, and further exacerbating tumor necrosis. Preclinical studies show that intratumoral injection of *C. novyi*-NT spores can significantly induce large-area necrosis of glioma, inhibit tumor progression, and preferentially colonize tumor tissues with extremely low colonization levels in normal brain tissue, demonstrating high safety (27). However, while monotherapy with *C. novyi*-NT can quickly clear the tumor core, it is often difficult to completely eradicate the tumor margin, which is less hypoxic. To address this, a preclinical study combined *C. novyi*-NT with liposomal doxorubicin, showing that this combination significantly improved survival benefits in glioma-bearing rats and effectively cleared residual surviving tumor cells at the tumor margin, indicating that combination with chemotherapy is a feasible strategy to further enhance efficacy (26).

In summary, the oncolytic therapy based on *C. novyi*-NT shows great potential in glioma treatment due to its unique hypoxia targeting and safety characteristics. However, it must be pointed out that *Clostridium* is an obligate anaerobe, and its germination and proliferation are mostly limited to the hypoxic necrotic area inside the tumor. Lacking active chemotactic movement ability, it struggles to penetrate the oxygen-rich peripheral tumor area. This spatial limitation makes it difficult to effectively clear the diffusely infiltrating surviving cells at the glioma margin and postoperative minimal residual disease. Therefore, while *Clostridium* monotherapy can effectively clear the tumor core, the risk of peripheral recurrence remains high (26, 28). Future development directions may not be limited to monotherapy but will focus on organically combining its precise tumor core oncolytic effect with chemotherapy, targeted therapy, or immunotherapy. Additionally, using imaging techniques for non-invasive monitoring of the treatment process is expected to provide key support for achieving personalized precision treatment (29).

### 3.3 Other potential strains

*Listeria monocytogenes* is a facultative intracellular parasitic bacterium. Due to its unique intracellular life cycle and powerful immune activation ability, it has become an important vector for tumor immunotherapy. After being taken up by antigen-presenting cells, *Listeria* secretes listeriolysin O (LLO) to disrupt the phagosomal membrane and enter the cytoplasm. The delivered tumor antigens can be presented via both MHC-I and MHC-II pathways, thereby efficiently activating CD8^+^ cytotoxic T cells and CD4^+^ helper T cells. Furthermore, *Listeria* vaccines can remodel the tumor microenvironment: on one hand, promoting the infiltration of IFN-γ^+^ effector CD8^+^ T cells and enhancing NK cell activity; on the other hand, reducing immunosuppressive myeloid-derived suppressor cells (MDSCs) and regulatory T cells (Tregs), transforming “cold” tumors into “hot” tumors (30, 31).

Strains like *Escherichia coli* and *Bifidobacterium* shows distinct therapeutic potential in glioma treatment. *E. coli* has a mature genetic manipulation system and controllable endotoxin. It can express therapeutic proteins such as TRAIL apoptosis-inducing ligand and IL-12 cytokine through gene editing, achieving significant tumor suppression in orthotopic glioma models. It can also cross the BBB via the “Trojan horse” mechanism using immune cells, improving brain delivery efficiency (32). *Bifidobacterium* is a common probiotic in the gut. It primarily inhibits glioma cell proliferation by blocking the activation of the MEK/ERK signaling pathway. It can also regulate gut microbiota composition, alter blood metabolite levels, and downregulate the expression of PD-1/PD-L1 immune checkpoint molecules on tumor cell surfaces, thereby enhancing the body’s antitumor immune response. These findings suggest that *Bifidobacterium* may serve as a potential adjuvant strategy for glioma treatment (33).

### 3.4 Personalized selection of oncolytic bacteria based on tumor microenvironment molecular subtypes

The degree of hypoxia, immune status, and metastatic characteristics vary significantly among different gliomas. Various oncolytic microorganisms have different natural colonization characteristics, applicable scenarios, and limitations. **Table 1** summarizes the basic chassis characteristics, compatible tumors, and inherent limitations of five mainstream oncolytic bacteria, providing a reference basis for personalized strain selection for tumors (34–36).

**Table 1:** Basic chassis characteristics, compatible tumors, and inherent limitations of mainstream oncolytic bacteria.

Strain category	Core biological characteristics	Compatible tumor microenvironment subtype	Inherent application limitations
*Salmonella* typhimurium	Facultative anaerobe, strong motility, mature type III secretion system, well-developed genetic editing tools	Solid tumors with moderate hypoxia, rich vasculature, and systemic micrometastases	Prone to significant clearance by the hepatic and splenic reticuloendothelial system after intravenous infusion; wild-type strains risk organ colonization and bacteremia, requiring construction of purine auxotrophic attenuated strains to limit off-target proliferation
*Clostridium novyi*	Obligate anaerobic spore-forming bacterium, spores germinate only in low-oxygen microenvironment, secretes phospholipases and hemolysins to destroy tumor cell membranes	Core necrotic areas of solid tumors with severe hypoxia and chemoradiotherapy resistance	Lacks autonomous movement, colonization limited to necrotic areas, unable to clear oxygen-rich peripheral infiltrating tumor cells; requires intratumoral injection of spores
*Listeria monocytogenes*	Facultative intracellular bacterium, secretes LLO to disrupt phagosomal membrane, can infect MDSCs and utilize them for migration to tumors	Solid tumors with poor T cell infiltration and immunosuppressive phenotype; suitable for tumor antigen vaccine delivery	Simple intratumoral administration recruits Ly6G^+^ neutrophils differentiated into PMN-MDSCs, inhibiting antitumor T cell function; risk of delayed systemic infection in immunocompromised individuals
*Escherichia coli* Nissle 1917	Gut commensal probiotic, highly modular genetic modification, naturally non-pathogenic	Suitable for oral adjuvant therapy of various solid tumors, especially combined with immune checkpoint inhibitors	Weak natural tumor targeting ability, lacks direct potent oncolytic effect, relies on artificial genetic modification to enhance efficacy
*Bifidobacterium*	Obligate anaerobic gut commensal, naturally tends to colonize hypoxic tumor tissue, well-tolerated in humans	Gastrointestinal tumors, late-stage palliative immune adjuvant therapy	Lacks genetic manipulation tools, difficult to stably express large therapeutic proteins, weak single-agent antitumor activity

### 3.5 Biomarker-guided personalization of oncolytic bacterial therapy

Biomarker-guided patient screening is expected to become an important approach for precision bacterial therapy in glioblastoma. A clinically applicable evaluation system should integrate at least four categories of biomarkers: (1) spatial hypoxia and necrosis indicators, including hypoxia-related imaging features, hypoxia-inducible factor-associated transcriptional programs, lactate accumulation, and perfusion characteristics (37); (2) immune microenvironment markers, encompassing CD8^+^ T cell infiltration levels, microglia or macrophage polarization status, regulatory T cell or myeloid-derived suppressor cell abundance, interferon signaling pathways, and cytokine profiles; (3) tumor cell molecular features, such as EGFRvIII, IL-13Rα2, mesenchymal transition traits, and DNA repair or therapy resistance-associated programs (38); and (4) host-related factors, including baseline inflammatory status, host antibacterial immunity, glucocorticoid use history, and immunosuppression levels (39).

This stratified framework can guide the selection of bacterial carriers and therapeutic payloads. For example, tumors with a high degree of hypoxia and necrosis are more suitable for obligate anaerobic spore-forming bacteria, whereas tumors with relatively accessible infiltrative margins are better suited for facultative anaerobes, localized delivery systems, or combination regimens. Immunologically “cold” glioblastomas may be treated with engineered bacteria capable of releasing cytokines or locally delivering checkpoint-blocking molecules, whereas patients with significant systemic immunosuppression require safer bacterial containment designs and more cautious dose escalation.

Using engineered Escherichia coli Nissle 1917, researchers designed bispecific engagers simultaneously targeting EGFRvIII and IL-13Rα2 based on the antigenic profile of glioblastoma. In a GBM mouse model, this personalized strategy achieved an 83% survival rate at 120 days, compared to approximately 17% in the single-target control group. This study demonstrates that tailoring bacterial payloads according to tumor molecular features can effectively overcome the heterogeneity barrier, providing an important proof of concept for personalized bacterial therapy (38).

## 4 Core molecular mechanisms of oncolytic bacteria in treating malignant glioma

### 4.1 Direct oncolysis and nutrient deprivation effect

After colonizing the glioma lesion, oncolytic bacteria can directly kill tumor cells through multiple pathways including metabolic competition, toxin lysis, vascular blockade, and oxidative stress, destroying tumor cell survival conditions from physical, metabolic, and oxidative dimensions, and clearing drug-resistant lesions in hypoxic areas.

#### 4.1.1 Metabolic starvation killing mediated by nutrient competition

Bacteria proliferate massively in the tumor hypoxic area, continuously competing for living space and consuming essential metabolic substrates for tumor cell proliferation, such as glucose, amino acids, and glutathione, causing metabolic deprivation in tumor cells, blocking the cell cycle, and inducing growth arrest. The deep hypoxic niche of glioma is the main habitat for tumor stem cells, where nutrient supply is already insufficient. The metabolic competition of bacteria can further limit the self-renewal ability of stem cells, weakening the potential for tumor recurrence at the source (40). On this basis, genetic engineering further enhances the nutrient deprivation ability of bacteria. Researchers overexpressed L-methioninase in attenuated *Salmonella*, constructing a tumor-localized methionine deprivation system. This engineered bacterium specifically colonizes tumor tissue and continuously degrades methionine, achieving significant oncolytic effects and almost completely inhibiting metastasis in various solid tumor models (41). Similarly, engineering *E. coli* to express glucose dehydrogenase increased its glucose consumption rate by nearly 1.4 times. By continuously depriving tumor-localized glucose, it effectively triggered pro-death autophagy and apoptosis in tumor cells (42). This metabolic starvation strategy based on nutrient competition can be enhanced through genetic engineering to increase the bacteria’s ability to consume metabolic substrates, achieving multi-level killing of the tumor core and infiltrative areas. The above-mentioned metabolic competition mechanism is shared among multiple solid tumors, but it is particularly important for GBM, as glioma stem cells enriched in the hypoxic niche are highly dependent on glycolysis, and this strategy can effectively eliminate the source of recurrence that is resistant to chemoradiotherapy.

#### 4.1.2 Bacterial toxin-induced cell lysis and death

During proliferation, oncolytic bacteria continuously secrete metabolic toxins. After cell lysis, they release active substances like lipopolysaccharides, which disrupt the integrity of tumor cell membranes, interfere with intracellular proliferation-related signaling pathways, and directly induce tumor cell lytic necrosis and apoptosis (43). Among the toxin family, pore-forming toxins (PFTs) are the most extensively studied. They assemble transmembrane pores in the target cell membrane, leading to ion imbalance and osmotic pressure changes, ultimately causing cell lysis or programmed cell death (44). Taking ClyA (cytolysin A) as an example, this toxin induces a mixed cell death mode of apoptosis, pyroptosis, and necrosis through a cholesterol-dependent mechanism, and releases damage-associated molecular patterns to activate antitumor immunity (45). In malignant glioma animal models, engineered *Salmonella* Typhimurium carrying ClyA specifically colonizes tumor tissue, induces large-area necrosis, inhibits tumor growth, and significantly prolongs survival (46). Furthermore, the pore-forming toxin listeriolysin O secreted by *Listeria monocytogenes* can trigger inflammatory lysis of tumor cells by activating the Gasdermin C-dependent pyroptosis pathway and promote dendritic cell maturation and CD8^+^ T cell infiltration (47).

For hypoxic drug-resistant cells that are difficult to clear with traditional chemoradiotherapy, obligate anaerobes can deeply colonize the necrotic core, relying on toxins to continuously lyse residual tumor tissue and reduce minimal residual disease (48). The *Clostridium* genus has a long history of application in this regard. Intravenous injection of *Clostridium* spores can germinate and proliferate in the hypoxic area of glioma, transforming the necrotic core into a liquefied abscess (49). Currently, synthetic biology techniques allow precise spatiotemporal regulation of toxin expression, using exogenous inducers to “activate” toxin expression on demand after bacterial colonization of the tumor, enhancing treatment precision and reducing off-target damage. Additionally, some bacterial toxins have immunomodulatory functions independent of pore formation, inducing tumor cell pyroptosis by activating pathways like the NLRP3 inflammasome (44), more effectively breaking the immunosuppressive state of the glioma microenvironment.

However, the clinical application of toxin therapy still faces challenges-how to balance the killing efficacy of toxins with neurological safety, and how to overcome inflammatory brain edema caused by bacterial entry into the brain after BBB disruption, remain core issues to be resolved in bacterial therapy for glioma.

#### 4.1.3 Intratumoral vascular targeting embolization for ischemic killing

Oncolytic bacteria can achieve ischemic killing through intratumoral vascular targeting embolization, a mechanism distinct from traditional anti-angiogenic drugs. Taking *Salmonella* YB1 as an example, after intravenous injection, bacteria passively retain in the irregular structures of tumor blood vessels, such as “shoulder structures” and “labyrinth structures.” Prolonged contact with endothelial cells induces local thrombosis, selectively blocking tumor blood supply and causing hypoxia; the hypoxic environment further activates bacterial proliferation, forming a positive feedback loop of “embolization-hypoxia-amplification-killing” (50). The highly tortuous, leaky, and structurally disordered blood vessels of glioblastoma precisely provide a structural basis for this physical retention (51). Compared with anti-VEGF drugs like Bevacizumab, which are prone to inducing vascular pattern switching leading to resistance, oncolytic bacteria directly destroy vascular structures through physical embolization, potentially circumventing such issues (51). This physical embolization effect holds particular value in GBM-the presence of the blood–brain barrier renders systemic anti-angiogenic agents poorly efficient in entering the brain, whereas local bacterial colonization can bypass this obstacle and act directly on intratumoral abnormal vasculature. However, whether this strategy can achieve stable and durable embolization effects in the highly vascularized and heterogeneous GBM requires further verification (50).

#### 4.1.4 ROS-mediated oxidative stress cytotoxicity

Oncolytic bacteria induce oxidative stress locally in the tumor, which is a key mechanism for their antitumor effects. Unlike traditional treatments, bacteria continuously produce reactive oxygen species (ROS), which can simultaneously trigger multiple cell death pathways, creating a “dual” or even “multi-pronged” killing effect.

On one hand, high concentrations of ROS can directly destroy tumor cells. Studies show that oncolytic bacteria can disrupt the metabolic homeostasis of tumor cells, causing a burst of ROS production, ultimately leading to tumor cell energy depletion and death (52). Another strategy combines bacteria with nanocatalytic materials, using endogenous hydrogen peroxide produced by bacteria to convert it into more toxic ROS species, thereby simultaneously activating multiple death pathways such as apoptosis and ferroptosis, achieving multi-angle killing and helping to circumvent resistance caused by single pathway blockade (53). On the other hand, ROS can synergistically enhance other treatment modalities. For example, ROS can induce the formation of autophagosomes in tumor cells, providing a favorable microenvironment for the replication of oncolytic viruses, thereby amplifying viral replication and oncolytic efficacy (54).

However, glioma cells are not passive recipients of oxidative damage. They often upregulate various antioxidant enzymes and the glutathione system to neutralize ROS, establishing a strong defense barrier and resisting treatment (55). This adaptive feature essentially reflects the systemic stress adaptation capability that GBM cells have evolved under multiple pressures, including hypoxia, nutrient deprivation, and endoplasmic reticulum stress-tumors rely on heat shock proteins to maintain proteostasis, the glutathione system to buffer oxidative damage, and the unfolded protein response (UPR) to cope with ER stress, with these three mechanisms synergistically constituting a “survival firewall” against therapy. Therefore, the success of oncolytic bacterial strategies depends not only on the “quantity” of ROS produced, but also on whether the tumor’s endogenous antioxidant capacity can be effectively weakened. An ideal strategy should simultaneously generate ROS and deplete the local antioxidant reserves of the tumor, achieving a “both offensive and defensive” therapeutic effect.

Based on this rationale, the stress adaptation mechanisms of tumors provide highly promising targets for the construction of engineered bacterial therapeutic platforms. By introducing tumor microenvironment-responsive promoters and genetic circuits, bacteria can be programmed to preferentially activate therapeutic effector molecules within glioblastoma tissues: engineered bacteria can be designed to express regulatory molecules targeting heat shock proteins, thereby interfering with tumor cell proteostasis and antigen presentation while inducing immunogenic cell death; they can deliver glutathione-depleting enzymes or NRF2 pathway inhibitors to dismantle the tumor’s oxidative defense system, rendering it more susceptible to ROS-mediated cytotoxicity; and they can express regulatory elements targeting the UPR pathway to weaken GBM cell tolerance to ER stress, thereby enhancing their sensitivity to radiotherapy, chemotherapy, and immune killing (56). Multiple preclinical studies have confirmed that bacteria and bacteria-derived delivery systems targeting the above stress pathways exhibit antitumor activity in GBM models, with some studies showing further efficacy enhancement when combined with conventional or immunotherapies (57).

### 4.2 Remodeling the tumor microenvironment and reversing the immunosuppressive state

#### 4.2.1 Systemic antitumor immune activation

After oncolytic bacteria lyse tumor cells, they release damage-associated molecular patterns (DAMPs) such as calreticulin, ATP, and HMGB1, along with a large amount of tumor-specific antigens, forming an endogenous tumor vaccine. These antigens are recognized, taken up, and presented by dendritic cells, effectively activating adaptive antitumor immunity mediated by CD4^+^ helper T cells and CD8^+^ cytotoxic T cells (58).

Furthermore, pathogen-associated molecular patterns (PAMPs) carried on the bacterial cell wall, such as peptidoglycan and lipopolysaccharide, can activate the host’s systemic innate immunity through Toll-like receptor pathways, stimulating the release of a large number of pro-inflammatory cytokines at the lesion site, recruiting a large number of immune effector cells such as neutrophils, peripheral-derived macrophages, and NK cells to infiltrate the tumor tissue, and downregulating the proportion of immunosuppressive cells such as Tregs and MDSCs, reversing the tumor immunosuppressive microenvironment at a systemic level (59). This immune activation effect can not only clear the local primary tumor lesion but also induce long-term systemic antitumor immune memory, effectively killing systemic micrometastases and residual tumor stem cells, achieving long-term tumor control (60). Although this effect is shared among multiple solid tumors, it is particularly critical for GBM as an immunologically “cold” tumor, as it can break the immune-privileged state of the brain and induce CD8^+^ T cells to cross the blood–brain barrier and infiltrate the lesion.

#### 4.2.2 Central nervous system-specific immune regulation

After crossing the BBB through multiple pathways and colonizing the glioma lesion, oncolytic bacteria can further regulate the unique immune network of the central nervous system, breaking the brain’s natural immune privilege, demonstrating intracranial tissue specificity. PAMPs carried by bacteria can be recognized by Toll-like receptors (TLRs) on microglia and infiltrating macrophages, driving their conversion from the M2 to the M1 antitumor phenotype. This polarization reversal not only restores the direct clearance ability of phagocytes against tumors but also improves the local antigen presentation environment, providing necessary conditions for T cell activation (61–63).

After colonizing the tumor hypoxic area, bacteria can significantly reduce the proportion of intracranial Tregs and MDSCs (18, 64). This effect may be related to changes in the chemokine profile-such as upregulation of CXCL10 facilitating CD8^+^ T cell recruitment, while downregulation of CCL2 reduces MDSC recruitment (63). By alleviating the immunosuppressive burden in the microenvironment, the antitumor activity of effector T cells can be released.

### 4.3 Overcoming the blood-brain barrier obstacle and efficient drug delivery

The BBB is the core physical bottleneck for glioma drug therapy. Oncolytic bacteria possess multiple mechanisms for crossing the barrier, effectively solving the problem of low drug entry efficiency into the brain. On one hand, some strains have natural barrier-crossing abilities. For example, *Listeria monocytogenes* and *E. coli* K1 can directly cross the BBB by invading host endothelial cells, receptor-mediated transport, etc. (65). On the other hand, bacteria can utilize the “Trojan horse” mechanism, being phagocytosed by peripheral blood monocytes or neutrophils and then entering the brain tumor tissue along with the immune cells’ trans-barrier migration, evading the interception effect of the BBB (66).

Meanwhile, the blood-tumor barrier formed by glioma growth disrupting the integrity of the BBB has significantly increased permeability, providing a natural window for bacterial enrichment. Combined with auxiliary technologies such as nanoparticle encapsulation and ultrasound-mediated barrier opening, the efficiency of bacterial colonization in the brain can be further improved. Furthermore, engineered bacteria can serve as living vectors, carrying chemotherapeutic drugs, therapeutic genes, and immunomodulators precisely into the brain, releasing therapeutic components locally at the tumor site, achieving targeted precision therapy, and significantly reducing the toxic side effects of systemic administration (67).

**Figure 2:**
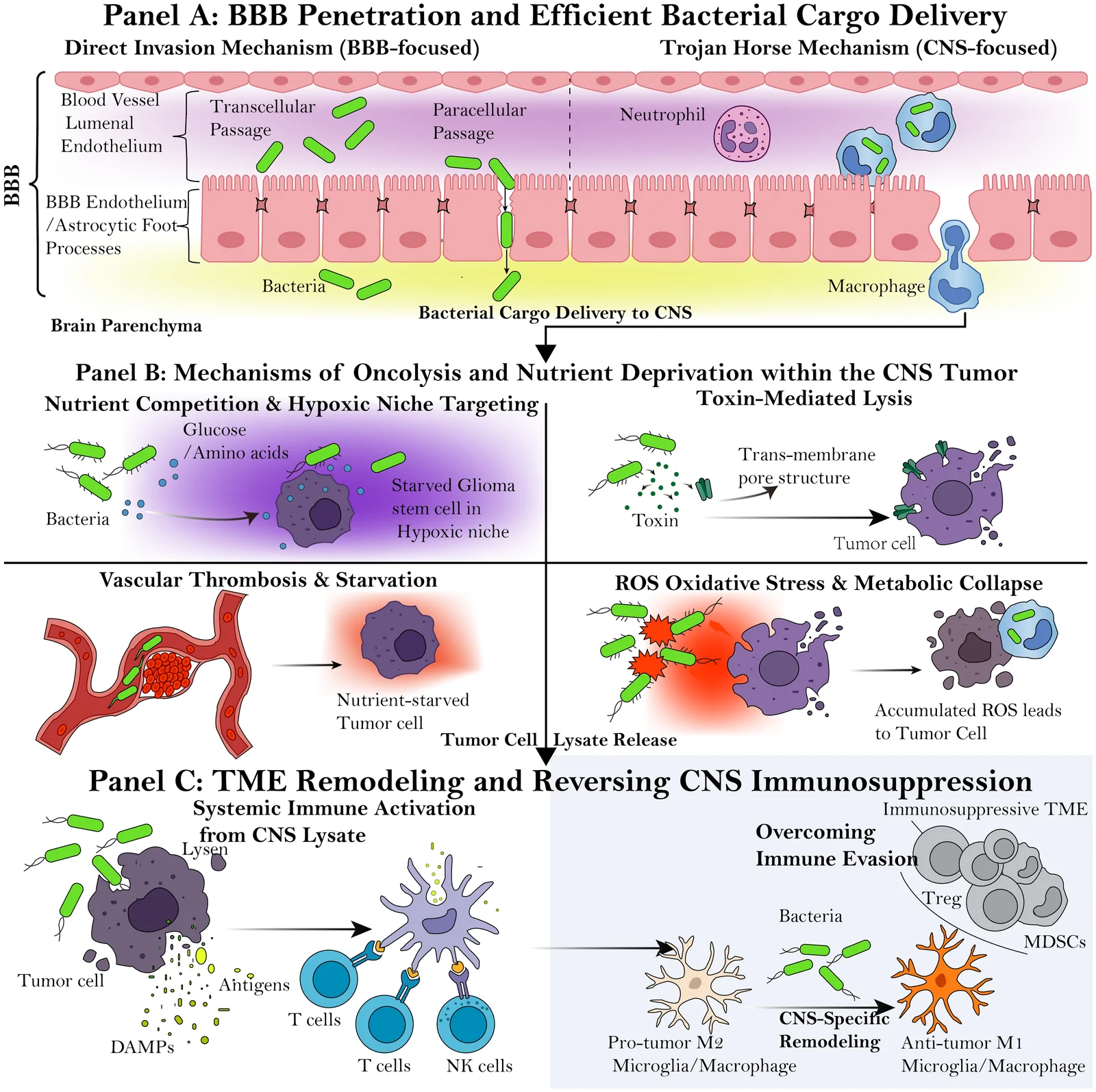
Multimodal therapeutic mechanisms of engineered oncolytic bacteria against glioblastoma. This schematic systematically depicts the sequential therapeutic cascade of bacteria targeting intracranial glioblastoma, divided into three interrelated panels:(1) Panel **(A)** Two core delivery pathways enabling blood-brain barrier penetration;(2) Panel **(B)** Four direct tumoricidal routes including nutrient deprivation, toxin-mediated lysis, vascular thrombosis and ROS-induced oxidative stress;(3) Panel **(C)** Immune activation mechanisms to remodel the tumor microenvironment and reverse central nervous system immunosuppression.

Two distinct pathways enabling bacteria to traverse the blood-brain barrier, as well as downstream oncolytic and immune remodeling effects, are illustrated (**Figure 2**).

## 5 Optimization strategies using genetic engineering and synthetic biology

Wild-type oncolytic bacteria have defects such as unstable virulence, insufficient targeting precision, and uncontrollable immune responses, making them unsuitable for direct clinical application. Relying on genetic engineering and synthetic biology technologies, it is possible to achieve strain attenuation for safety, precision targeting, and intelligent functionalization, which are core technical supports for promoting clinical translation.

### 5.1 Strain attenuation and safety optimization

Safety is the primary prerequisite for the clinical application of oncolytic bacteria, and precise gene attenuation is the core modification method. By knocking out bacterial virulence genes and toxin synthesis genes, the pathogenicity of the strain can be eliminated while retaining tumor colonization and oncolytic activity. For example, knocking out the *msbB* gene in *Salmonella* modifies the lipid A structure, reducing the risk of cytokine storms caused by endotoxins; knocking out the *purI* gene creates a purine auxotrophic strain that can only survive in the tumor’s high-purine microenvironment, further enhancing tumor targeting (68). Knocking out the alpha-toxin gene in *Clostridium novyi* completely eliminates potent toxic damage while retaining hypoxic lytic ability (69). Simultaneously, introducing biological containment systems such as “suicide genes” or “non-natural amino acid-dependent circuits” ensures that bacteria rapidly die after leaving the tumor microenvironment, preventing systemic infection and ectopic colonization risk, constructing multiple safety barriers (70). A study constructed liquid nitrogen-frozen shock macrophage carriers to encapsulate VNP20009. The macrophage shell shields the bacterial immune antigens, reducing peripheral immune hyperactivity and liver damage. The freezing treatment only eliminates the proliferation risk of macrophages without affecting the activity of intracellular *Salmonella*, which is slowly released and colonized upon reaching the tumor, balancing targeted enrichment and *in vivo* biocompatibility, forming a new safety protection idea at the delivery level (71).

### 5.2 Targeting enhancement and functional gene loading

Through genetic modification, the glioma targeting ability of bacteria can be enhanced, while simultaneously endowing them with diversified therapeutic functions.

In terms of targeting enhancement, besides modifying bacterial surface proteins to specifically bind to highly expressed antigens in glioma such as EGFRvIII and IL-13Rα2 (72), using dual-targeting or multi-targeting recognition element designs can effectively address recognition escape caused by tumor antigen heterogeneity. Simultaneously expressing binding molecules targeting different tumor antigens in the same strain enables broad-spectrum recognition and labeling of heterogeneous tumor cell populations, avoiding treatment failure due to the loss of a single antigen (38). Furthermore, using promoters responsive to characteristic signals of the tumor microenvironment (such as hypoxia, low pH) to regulate the expression of functional genes can strictly confine the release of therapeutic components to the tumor site, reducing systemic exposure risk (36).

Once the bacteria successfully colonize the tumor site, their large genome capacity advantage can be fully utilized. By loading multiple therapeutic genes, a “one bacterium, multiple effects” therapeutic outcome can be achieved. Specifically, cytokines like IL-12 and GM-CSF can be expressed to amplify antitumor immune responses; pro-apoptotic proteins like TRAIL and BID can be expressed to directly induce tumor cell apoptosis; single-chain antibodies against PD-1 and TIGIT can be expressed to achieve local immune checkpoint blockade, synergistically enhancing immunotherapy efficacy; cytosine deaminase can be expressed to mediate prodrug conversion, constructing a targeted chemotherapy system (73). On this basis, further research has expanded into the following directions: expression of bispecific engagers can direct immune effector cells to the tumor periphery, enhancing immune synapse formation and killing efficiency (38); using modular gene assembly tools, multiple functional genes can be simultaneously integrated into the bacterial genome, such as loading both immune checkpoint inhibitor and pro-inflammatory cytokine encoding genes, relieving immunosuppression while activating effector cell function, achieving synergistic effects (38, 74). Additionally, the concept of functional gene loading can be extended to bacteria-derived carriers such as bacterial ghosts or outer membrane vesicles. These carriers retain the targeting molecules and immune adjuvant properties of the parent bacteria while possessing higher biosafety and drug loading capacity, efficiently carrying therapeutic nucleic acids or chemical drugs, forming a safe, controllable, and multifunctional delivery platform (74).

### 5.3 Precise regulation of intelligent gene circuits

Synthetic biology gene circuits can achieve spatiotemporal regulation of bacterial behavior, creating programmable “living intelligent drugs.” Lysis circuits based on quorum sensing systems are constructed. When the bacterial density within the tumor reaches a threshold, the lysis program is automatically activated, releasing therapeutic drugs in a pulsatile manner, avoiding local inflammatory damage caused by excessive bacterial proliferation while continuously supplying therapeutic components (75). This population-level synergistic strategy, by dividing therapeutic functions among multiple strains, can achieve the programmed and sequential release of multiple drugs without increasing the metabolic burden on a single strain, thereby achieving spatiotemporal synergistic therapeutic effects (76).

Constructing microenvironment-responsive logic circuits, using characteristic signals of glioma such as hypoxia, low pH, and high lactate as triggers, precisely activates functions like oncolysis, immune activation, and drug release, achieving precise adaptation to the heterogeneous tumor microenvironment (36). However, a single microenvironment signal is not unique to tumors *in vivo*. To improve specificity, it is noteworthy that researchers have begun to construct “multi-input logic gates,” requiring bacteria to simultaneously sense two or more TME signals-such as the simultaneous presence of hypoxia and high lactate signals-to activate downstream killing programs. This “AND” logic design effectively avoids the “off-target” risk that a single microenvironment signal might pose in non-tumor tissues, providing a more reliable solution for addressing the heterogeneity within glioma (77).

A recent study further demonstrated the application potential of self-regulating genetic circuits in tumor-targeting bacterial therapy. Zou et al. engineered *Escherichia coli* Nissle 1917 to construct a lactate-sensing circuit that couples high lactate recognition with the expression of bacterial essential genes. This engineered strain can preferentially proliferate under lactate concentrations characteristic of the tumor microenvironment, while a quorum-sensing module regulates the local production of α-hemolysin. In addition, lactate-inducible coagulase expression promotes local thrombosis, thereby restricting bacterial and toxin leakage and enhancing tumor suppression. Although this study was conducted in a colorectal cancer tumor model rather than in glioblastoma, its design provides a highly valuable proof of concept for multi-layered signal sensing, therapeutic effector activation, spatial containment, and biosafety control. In glioblastoma research, analogous circuits could be engineered to integrate hypoxia and lactate signals; however, their actual efficacy must be validated in brain-specific models, as lactate distribution, blood–brain barrier integrity, and neuroinflammatory toxicity in the brain differ from those in peripheral tumors (78).

Building on the maturity of single regulatory circuits, multimodal gene circuits can integrate multiple functions such as photothermal therapy, immune regulation, and targeted drug delivery, achieving personalized precision treatment for glioma (79). In addition to utilizing internal tumor signals, responsiveness to external physical signals opens a new dimension for precise regulation. For example, combining a heat-sensitive transcriptional repressor with an ultrasound-controlled release system can achieve non-invasive, on-demand expression and release of therapeutic proteins in deep-seated lesions, leveraging the good tissue penetration of ultrasound (80).

Drug delivery should be regarded as an active biological decision network rather than a passive transport process (72). The design philosophy of the genetic circuits described above essentially represents a systematic logic programming of bacterial therapeutic behavior. At a more global level, the therapeutic output of oncolytic bacteria can be conceptualized as a closed-loop process driven by a sequence of sequential decisions: whether bacteria can survive, where they colonize, when they proliferate, which effector payloads they express, whether they undergo lysis, and how they respond to host immunity-all of which require precise spatiotemporal programming. This decision network can be stratified into four functional modules. The first module is environmental sensing, encompassing the perception of oxygen, lactate, pH, glucose, inflammatory cytokines, and tumor-associated metabolites. The second is spatial decision-making, determining whether bacteria remain within the tumor, invade tumor cells, or are cleared from normal tissues. The third is therapeutic effector output, covering toxin release, cytokine secretion, prodrug conversion, metabolic reprogramming, or viral activation. The fourth is termination and safety control, including quorum sensing-mediated lysis, auxotrophy-based regulation, suicide switches, antibiotic susceptibility, and exogenously inducible system shutdown (36, 68). This systematic stratified architecture is particularly critical for glioblastoma therapy-given the exceptional vulnerability of brain tissue, bacterial overactivation can trigger cerebral edema, elevated intracranial pressure, neuroinflammation, and even irreversible neurological damage. Therefore, future bacterial therapeutic formulations must be designed as closed-loop systems that integrate sensing, decision-making, effector execution, monitoring, and termination to achieve both safety and efficacy. Building upon this programmability of bacterial behavioral logic, researchers have further extended this concept to the dimension of therapeutic “spatial coverage” by constructing an engineered bacteria–oncolytic virus tandem delivery system, aiming to overcome the inherent limitations of locally restricted bacterial action.

### 5.4 Construction of engineered bacteria–oncolytic virus tandem delivery system

Although conventional engineered bacteria therapy can achieve tumor-targeted colonization, its direct oncolytic effect is limited to the bacterial enrichment area, making it difficult to cover the widely infiltrating tumor margins of glioma. To expand the therapeutic radius, researchers have constructed an engineered bacteria–oncolytic virus tandem delivery system-using attenuated *Salmonella* as an intracellular carrier for the viral genome. After the bacteria invade tumor cells, they initiate viral RNA transcription and release. The virus utilizes its own internal ribosome entry site to directly initiate translation and replication, completing the relay from “bacterial carriage” to “viral spread.” This strategy not only allows bacteria to evade the neutralizing effect of circulating antibodies, safely delivering the viral genome into the tumor, but also utilizes the virus’s diffusion ability to compensate for the local limitations of bacteria (79). Although this strategy has currently been validated in non-CNS tumors, its design logic-using bacteria to cross the BBB to target infiltrative tumors and locally spreading oncolytic viruses to expand the killing range-provides a highly promising engineered solution for preventing postoperative recurrence of glioma.

### 5.5 Programmable oncolytic virus backbones based on genetic logic gates and the CRISPR system

The engineered bacteria–oncolytic virus tandem delivery system described above addresses the safe delivery of viruses; however, the precise action of viruses after entering the tumor still relies on the programmability of the backbone itself. In recent years, the convergence of synthetic biology and gene-editing technologies has upgraded oncolytic viruses from “natural lytic tools” to “intelligent therapeutic platforms.”

Genetic logic gates, drawing on the “AND gate” principle from electronic engineering, require viral replication to be activated only in the presence of two or more tumor-specific signals simultaneously, thereby greatly enhancing targeting precision. Researchers have constructed synthetic super-enhancers that recognize the regulatory network of GBM stem cells to drive the expression of viral therapeutic genes, restricting viral lysis strictly to tumor cells that simultaneously satisfy multiple molecular criteria and effectively avoiding off-target damage to normal neural cells (81).

The CRISPR-Cas9 technology further empowers viral backbones through two mechanisms: resistance circumvention and *in situ* editing. Genome-wide CRISPR screens have identified BRD9 as a key factor in GBM resistance to oncolytic HSV-1, and its knockout or inhibition significantly enhances viral replication and immunogenic cell death (82); CRISPR metabolic screens have also identified negative immune regulatory targets such as PLCE1 (83). At the direct application level, oncolytic viruses can be engineered to carry CRISPR components targeting PD-1/CTLA-4, enabling *in situ* knockout of immunosuppressive genes locally within the tumor, thereby achieving a triple function of “lysis–activation–de-repression” with editing confined to the virus-infected area, thus avoiding systemic risks (84).

The integration of logic gates and CRISPR synergistically tackles two major challenges in GBM therapy: logic gates enhance tumor recognition precision through multiplex signal verification and reduce off-target toxicity, while virus-encoded CRISPR components locally remodel the immune microenvironment, with editing activities restricted by logic gates to initiate only within the tumor region, avoiding systemic immunosuppression (85). This programmable viral backbone complements the aforementioned tandem delivery system, together constituting a complete engineering framework for oncolytic microbial therapy in GBM.

### 5.6 Biomaterial composite modification strategies

Sole oncolytic bacteria therapy faces many limitations *in vivo* applications, including rapid clearance by the immune system, difficulty crossing the BBB, insufficient tumor targeting efficiency, and potential toxic side effects. To overcome these bottlenecks, researchers have combined biomaterials with bacteria or bacterial derivatives to form multifunctional delivery systems, significantly improving the *in vivo* stability, targeting, and therapeutic efficacy of bacterial preparations. The construction of bacteria–nanomaterial hybrids is an important direction of this strategy. Using the specific ABC transporter pathway of bacteria, nanoparticles can be efficiently internalized into the bacterial cytoplasm, forming a so-called “Trojan bacteria” system. This strategy retains the bacteria’s ability to actively target tumors and cross the BBB while using nanomaterials to achieve additional functions such as photothermal conversion and imaging tracing (86). Compared to loading nanomaterials on the bacterial surface, intracellular loading better maintains the integrity of the bacterial envelope, which is beneficial for long-term survival and barrier crossing *in vivo*.

Bacterial membrane biomimetic modification represents another important strategy. By extracting outer membrane vesicles from engineered bacteria or directly using bacterial membranes to coat nanodrug cores, a delivery platform combining the natural targeting properties of bacteria with the high drug loading capacity of nanomedicines can be constructed. On this basis, displaying targeting peptides such as Angiopep-2, chlorotoxin, and cell-penetrating peptides on the bacterial membrane surface through genetic engineering can further enhance their ability to penetrate the BBB and actively recognize glioma cells (57, 87). Furthermore, the bacterial membrane itself is rich in PAMPs, which can activate innate immune pathways while delivering drugs, exerting a “drug delivery–immune activation” dual function (57).

Bacteria–hydrogel composite systems have opened new avenues for postoperative local treatment. Loading engineered bacteria into injectable hydrogels and filling the tumor resection cavity can achieve sustained release and persistent colonization of bacteria in the local microenvironment, effectively addressing the problem of rapid drug clearance caused by cerebrospinal fluid flushing (18, 38). Some smart hydrogels can also respond to high concentrations of signaling molecules like ATP in the tumor microenvironment, releasing immune adjuvants on demand, further enhancing the intensity and durability of the local antitumor immune response (18).

With the deep integration of materials science and synthetic biology, composite modification strategies are expected to provide more mature technical support for the clinical translation of oncolytic bacteria in treating glioma.

**Figure 3:**
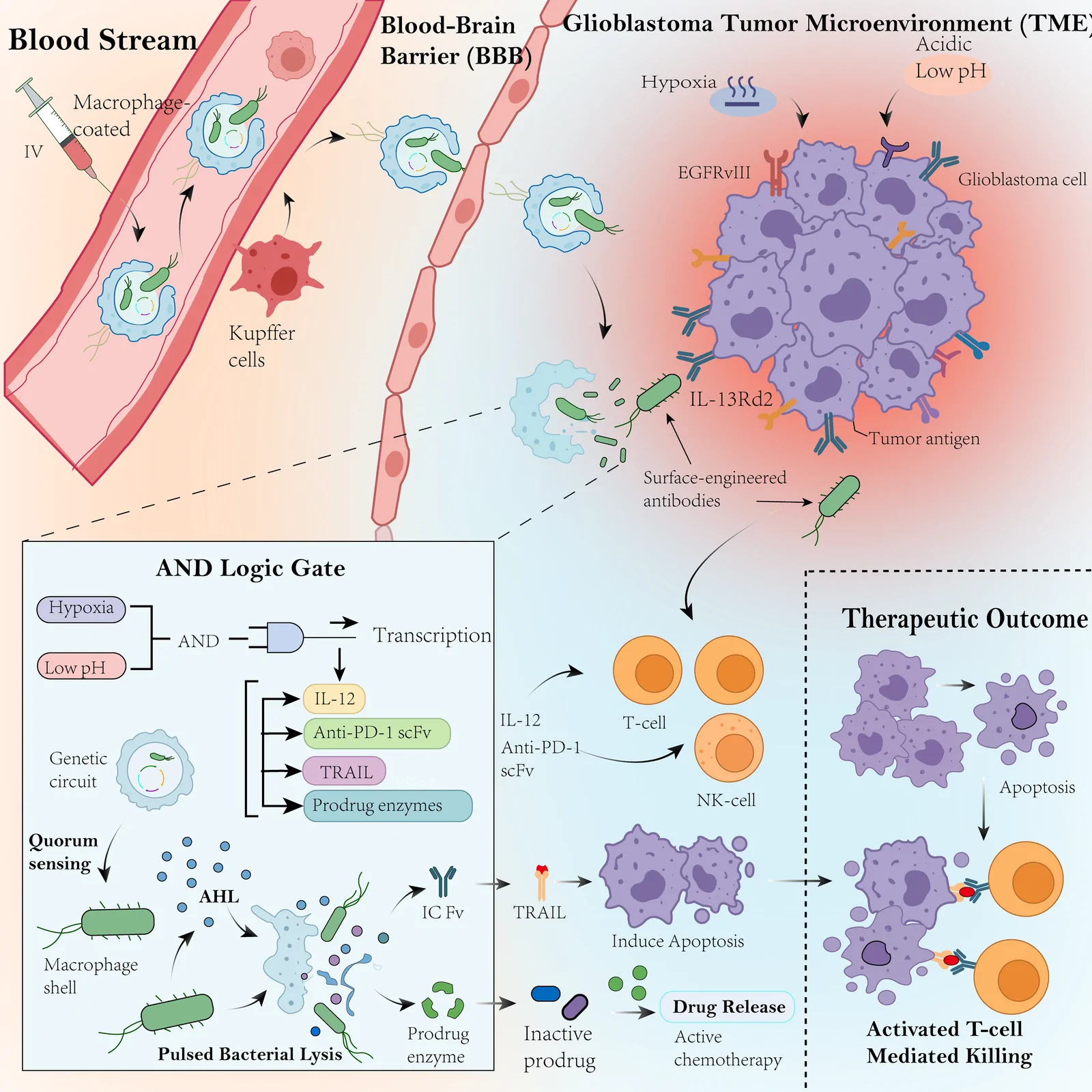
Synergistic therapeutic mechanisms of macrophage-camouflaged engineered bacteria for glioblastoma treatment. This diagram illustrates the integrated workflow of immune cell-mediated delivery, TME-responsive genetic circuits and *in situ* release of multifunctional therapeutic payloads: (1) Peripheral circulation: Macrophage shells shield bacteria for immune evasion and blood-brain barrier traversal; (2) Tumor microenvironment: Hypoxia/low pH dual-input AND logic gates control therapeutic gene transcription; (3) Tumor-killing stage: Triple synergistic anti-tumor effects including apoptosis induction, immune checkpoint blockade and prodrug conversion.

Tumor microenvironment-responsive AND logic gene circuits and macrophage-mediated bacterial delivery are elaborated in detail (**Figure 3**).

## 6 Preclinical research and clinical translation progress

### 6.1 Efficacy validation in preclinical models

The orthotopic glioma mouse model is the core preclinical system for evaluating the efficacy of oncolytic bacteria, realistically simulating the microenvironment, infiltrative growth characteristics, and BBB structure of human glioma. Numerous preclinical studies have confirmed that engineered strains like *Salmonella* and *Clostridium novyi* can precisely target orthotopic gliomas, significantly inhibit tumor proliferation, reduce tumor volume, and greatly prolong the survival of tumor-bearing animals (88). For example, intravenous injection of attenuated *Salmonella* carrying inducible cytolysin A specifically colonized advanced rat orthotopic gliomas, inducing central tumor necrosis and inhibiting peripheral proliferative zone invasion, extending the median survival from 29 days to 39 days (89); in a rabbit VX2 orthotopic brain tumor model, intratumoral injection of *Clostridium novyi* also led to significant tumor destruction (90). In temozolomide-resistant glioma models, oncolytic bacteria still exerted potent tumor suppression, effectively overcoming the bottleneck of chemotherapy resistance, demonstrating core value in compensating for the shortcomings of traditional treatments (91). Simultaneously, multimodal imaging monitoring confirmed that bacterial colonization was largely limited to the tumor lesion, with negligible abnormal colonization in normal brain tissue, and local inflammatory reactions were controllable, indicating good *in vivo* safety (92).

Combination therapy studies show that oncolytic bacteria combined with radiotherapy, chemotherapy, and immune checkpoint inhibitors produce significant synergistic effects, greatly enhancing tumor suppression beyond single-agent regimens (93). In combination with chemotherapy, bacteria can act as a “living drug factory,” continuously releasing prodrug-converting enzymes locally in the tumor, converting non-toxic prodrugs into active chemotherapeutic drugs, achieving *in situ* activation and precise delivery of drugs (94); simultaneously, the bacteria’s own tumor tropism allows them to penetrate hypoxic areas that conventional nanocarriers cannot reach, efficiently delivering chemotherapeutic drugs to the deep tumor (95). In combination with radiotherapy, bacteria can enhance radiosensitivity through multiple mechanisms: facultative anaerobes colonizing the tumor hypoxic area can exacerbate local hypoxia by consuming oxygen or act directly as radiosensitizers, making the originally radioresistant hypoxic area more sensitive (94); furthermore, bacteria can induce immunogenic cell death in tumor cells, triggering an “*in situ* vaccine” effect combined with radiotherapy, activating systemic antitumor immune responses (68). When combined with immune checkpoint inhibitors, oncolytic bacteria, through their inherent immunogenicity and locally produced immunomodulatory molecules, remodel the immunosuppressive “cold tumor” into a “hot tumor” infiltrated by T cells, creating favorable conditions for checkpoint inhibitors to work (74). This multi-dimensional synergistic design makes oncolytic bacteria an ideal platform connecting different treatment modalities.

It is noteworthy that the timing of administration in combination therapy has a decisive impact on efficacy, and the mechanism is far more complex than simple addition. Taking *Listeria monocytogenes* (Lm) as an example, simple intratumoral (IT) injection of Lm unexpectedly recruits Ly6G^+^ neutrophils and polarizes them into pro-tumor PMN-MDSCs, actually accelerating tumor growth. However, if intravenous Lm immunization is performed prior to intratumoral injection, it induces systemic anti-Lm CD8^+^ T cells. When subsequent IT Lm recruits bacteria to the tumor site, these specific T cells can clear the immunosuppressive PMN-MDSCs carrying bacteria. Simultaneously, through perforin-dependent killing and release of IFN-γ/TNF-α, they directly inhibit tumor cell proliferation and promote cross-presentation of tumor antigens, thereby transforming harmful bacterial colonization into a durable antitumor immune response (96). This finding suggests that combining oncolytic bacteria with immunotherapy is not a simple “drug stacking” but requires a rationally designed “pre-immunization–local activation” sequential strategy based on the characteristics of the bacterial strain and the host immune status.

### 6.2 Early clinical trial progress

Currently, clinical trials of oncolytic bacteria are mostly focused on solid tumors, with specific research on glioma still in the early exploratory stage. However, existing trial data provide key references for the clinical translation of glioma. Phase I clinical trials of attenuated *Salmonella* VNP20009 showed good safety after systemic administration, with main adverse reactions being mild flu-like symptoms and no serious complications such as severe sepsis or organ damage. However, the density of tumor colonization in humans was lower than in animal models, and the antitumor efficacy of the single strain was limited (97). The Phase I clinical trial of novyi-NT enrolled patients with refractory solid tumors, and intratumoral administration effectively induced tumor necrosis and local inflammatory responses, achieving shrinkage of some tumor lesions with good tolerability and controllable toxicity. Its Phase Ib study in combination with PD-1 inhibitors confirmed that bacterial therapy and immunotherapy have significant synergistic effects without increasing the risk of adverse reactions (48). The tumor vaccine CRS-207, which uses *Listeria monocytogenes* as a vector, has completed clinical validation in peripheral solid tumors such as pancreatic cancer and ovarian cancer, showing good safety but limited efficacy as monotherapy (2).

Safety exploration for brain tumors indicated that attenuated bacterial strains were strictly localized within tumor tissues and did not induce severe central nervous system complications such as meningitis or brain abscesses, establishing a safety foundation for clinical trials in glioma (98).

## 7 Core challenges and countermeasures for clinical translation

### 7.1 Biosafety control challenges

As live biotherapeutic products, the *in vivo* controllability of oncolytic bacteria represents the greatest barrier to clinical translation. In immunocompromised glioma patients, there are potential risks of bacterial escape from colonization, opportunistic infections, systemic inflammatory responses, and even sepsis; the dynamic process of bacterial proliferation and lysis *in vivo* is difficult to precisely regulate, potentially causing excessive local inflammation leading to central nervous system adverse effects such as brain edema and neurological damage; additionally, genetically engineered bacteria carry potential risks of horizontal gene transfer and environmental escape (99). Unlike conventional drugs that are gradually metabolized and cleared after entering the body, live bacterial formulations can autonomously replicate, persist, and even disseminate to non-target tissues, undergoing genetic and phenotypic variations. Even with attenuated strains, improper control of dosage, administration route, or payload expression may still induce systemic inflammation or off-target colonization (77). Therefore, safety control should not be limited to strain design alone but must integrate individualized dose gradient regimens and dynamic clinical monitoring systems into the overall strategy.

To address these issues, precise control of bacterial behavior *in vivo* can be achieved through the construction of multiple biosafety circuits, precise attenuation modifications, and the implantation of controllable suicide systems; optimizing strain formulations and administration doses to establish individualized dose gradient regimens; and improving clinical monitoring systems to track patient inflammatory markers and bacterial load in real-time, formulating emergency intervention protocols (100). Among these, auxotrophic design can strictly confine bacterial proliferation to the tumor microenvironment, while gene circuits such as suicide switches provide reversible means to eliminate uncontrolled bacterial proliferation (12, 77). Regarding the route of administration, intratumoral injection allows bacteria to reach high local concentrations while reducing systemic exposure risk (101).

Significant differences exist between preclinical studies and human trials. Taking the attenuated *Salmonella* strain VNP20009 as an example, this trial revealed a fundamental translational bottleneck: although this strain exhibited extremely high targeted colonization rates in tumor-bearing mice, after intravenous administration in humans, the bacteria were cleared from peripheral blood much faster than in rodents, resulting in only a few patients having detectable colonizing bacteria in tumors even at the maximum tolerated dose, with no objective tumor regression observed. The study explicitly pointed out that the rapid clearance of bacteria by the human immune system and the high sensitivity to LPS-related inflammatory factors are core obstacles to extrapolating animal data to clinical settings (102). This suggests that even with rigorously attenuated strains, their behavior in clinical applications remains difficult to fully predict, necessitating the establishment of a clinical risk management system covering pre- and post-treatment serum cytokine level detection, dynamic monitoring of bacterial load, and antibiotic intervention or emergency clearance procedures (12, 77).

Safety management of oncolytic bacteria as “living drugs” should not be confined to genetic attenuation at the strain level, but rather re-examined from a systems engineering perspective in terms of their design logic. The design of oncolytic bacteria for glioblastoma should follow a systems and adaptive engineering framework: at the systemic level, therapy must be viewed as an interaction process among the bacterial chassis, tumor ecosystem, host immune system, administration route, therapeutic payloads, and clinical interventions (68); modification of any single component may alter the behavior of the entire system-for instance, *Salmonella*-mediated metabolic interference, although capable of suppressing tumor metabolism, may also inadvertently impair T cell function due to nutrient competition (20). Therefore, safety optimization cannot rely on static strain modification, but should instead introduce an adaptive iterative cycle of “design–build–test–learn” (DBTL). Preclinical evaluation should incorporate multi-layered systems including patient-derived xenograft models, immunocompetent animal models, organoids, and microfluidic blood–brain barrier models, and therapeutic parameter adjustments should be guided by real-time feedback based on dynamic indicators such as bacterial load, cytokine profiles, radiographic changes, and immune cell composition. Only by upgrading safety management from “single-point control” to a “closed-loop system” can the potential risks of live bacterial formulations to the central nervous system be minimized while ensuring therapeutic efficacy.

### 7.2 Insufficient delivery efficiency and colonization stability

The residual barrier effect of the BBB and the clearance effect of the immune system lead to low intracranial colonization efficiency and poor stability after intravenous administration, representing a core technical issue for suboptimal efficacy. Although intratumoral injection can bypass the barrier, it cannot cover the infiltrative micro-foci of glioma, resulting in treatment blind spots (103). To objectively compare the pros and cons of different administration strategies, the following summarizes representative studies of current major administration routes from four dimensions: BBB penetration, colonization characteristics, efficacy evidence, and limitations (**Table 2**).

**Table 2:** Comparison of oncolytic bacteria administration routes.

Administration route	BBB penetration	Intratumoral distribution	Efficacy evaluation	Core limitations	Ref
Intravenous Injection	Passive extravasation via tumor vascular leakage; “Trojan horse”	Cover main tumor mass and distant micro-satellite foci;	Prolongs survival; OMV-C-C can induce CD8^+^ T cell-dependent antitumor immunity	Liver/spleen sequestration; limited by residual BBB	(26, 57, 104)
Intracranial Injection	Direct injection into tumor parenchyma, completely bypassing BBB	High local bacterial concentration; longer colonization duration than IV administration; significant effect on main tumor mass	Significantly inhibits tumor growth, prolongs survival;	nvasive procedure, only applicable to locally accessible lesions; cannot cover infiltrative micro-foci; clear dose tolerance upper limit	(105)
Postoperative Intracavitary Injection	Direct exposure to surgical cavity after resection, no need to cross BBB	Hydrogel confines bacteria to the surgical cavity, prolonging retention time; targets “satellite foci” and infiltrating tumor cells	Effectively activates innate and adaptive immunity,	Depends on tumor resection surgery; difficult for deep functional areas or unresectable recurrent patients	(18)
Oral Administration	activates systemic antitumor immunity via gut immune system	Bacteria act as DNA vaccine vectors, do not directly colonize the brain;	Induces specific T cell responses in recurrent GBM patients;	Antitumor efficacy requires large-scale clinical validation;	(106)
Nasal-Brain Pathway	Bypasses BBB via olfactory or trigeminal nerves, or enters brain after absorption through highly vascularized nasal mucosa	Limited delivery dose; specific intracranial colonization data lacking; still in preclinical mechanism exploration stage	No direct efficacy validation data in GBM models	Low delivery efficiency and difficult clinical translation; lacks GBM-specific validation	(104)

Currently, there is no unified standard for the administration of oncolytic bacteria in glioma therapy, with intravenous injection and intratumoral injection being the most commonly used routes, each with distinct applicable boundaries. Intravenous injection is simple to perform and repeatable, theoretically capable of covering microscopic lesions throughout the brain, yet it faces two major bottlenecks: bacteria are prone to substantial sequestration by the reticuloendothelial system of the liver and spleen during circulation, and the residual barrier effect of the blood–brain barrier further limits brain entry, resulting in intracranial colonization efficiency far lower than that predicted in animal models. Intratumoral injection bypasses the blood–brain barrier to achieve high local concentrations, with clear preclinical efficacy; however, its invasive nature makes it difficult to cover infiltrating microlesions, and it imposes higher demands on surgical tolerance in patients with recurrence. Convection-enhanced delivery (CED) utilizes continuous low positive pressure to drive drug convection and diffusion within the brain interstitium, bypassing the blood–brain barrier to achieve large-volume, homogeneously distributed *in situ* delivery, and has been validated for safety in clinical trials of other biologic agents for glioma. Focused ultrasound-mediated blood–brain barrier opening represents a non-invasive strategy that, in combination with microbubbles, enables reversible and localized barrier opening in specific brain regions, and when combined with bacteria–nanoparticle complexes, holds promise for spatiotemporally controllable bacterial entry into the brain (108).

In summary, the administration route for oncolytic bacteria should follow the principle of individualized stratification: for resectable primary lesions, postoperative intracavitary injection or sustained-release hydrogel implantation is the preferred option; for diffuse or multifocal drug-resistant lesions, intravenous injection combined with nano-camouflage and focused ultrasound-mediated barrier opening represents a direction for improvement; for deep-seated or recurrent lesions, precision interventional approaches such as convection-enhanced delivery can balance coverage and safety. Future optimization of delivery systems can be achieved by: modifying bacterial surface structures to enhance barrier-crossing ability; combining ultrasound, nanocarriers, and vascular normalization technologies to improve bacterial enrichment efficiency in the brain; and optimizing administration routes, exploring precise interventional delivery methods such as intra-arterial administration and convection-enhanced delivery, to balance lesion coverage and colonization stability (107).

### 7.3 Non-standardized efficacy evaluation system and lack of clinical data

Currently, there is no standardized efficacy evaluation system for oncolytic bacteria. Bacterial-induced tumor necrosis and tumor progression are difficult to distinguish in conventional imaging, leading to potential misjudgment of efficacy (108). Additionally, existing research is primarily based on preclinical animal experiments, lacking large-sample, multi-center, long-term follow-up clinical trial data specifically for glioma. The long-term safety, long-term efficacy, and recurrence prevention effects of bacterial strains remain unclear (109). It is necessary to construct a multi-dimensional efficacy evaluation system, combining molecular imaging, bacterial tracking technology, immune indicator detection, and survival analysis to achieve precise efficacy assessment; and accelerate the advancement of glioma-specific clinical trials to optimize core parameters such as administration regimens, combination therapy timing, and dose ratios, accumulating high-level evidence-based medical evidence (110).

### 7.4 Imperfect regulatory, ethical, and clinical application systems

Oncolytic bacteria possess dual attributes as both biological products and gene therapy products, subject to stringent regulatory standards. Currently, there is no globally unified standard for production quality control, clinical operation, and safety supervision (111). Unlike oncolytic viruses, which have established mature regulatory frameworks, oncolytic bacteria, as complex living organisms with autonomous metabolism, pose high technical barriers with their metabolic complexity and the CMC requirements for batch-to-batch consistency of live products. The strong immunostimulatory components carried by live bacteria, even in an attenuated state, may still trigger severe cytokine storms (112). Furthermore, public awareness of “bacterial cancer therapy” is insufficient, leading to psychological resistance, and the application of genetically modified microorganisms for intracranial treatment raises special ethical considerations, particularly regarding the safety and long-term effects in pediatric glioma patients (113). In the future, it is necessary to accelerate the establishment of standardized bacterial strain production and quality control systems, improve ethical review and refine informed consent; promote the integration of AI-driven gene circuit design with multimodal *in vivo* imaging technology to construct a closed-loop “design-monitor-control” system, providing technical support for regulatory approval (112); and strengthen academic science communication to enhance acceptance among clinicians and the public (114).

## 8 Controversies and unresolved issues

Although oncolytic bacterial therapy for malignant glioma has shown considerable promise in preclinical studies, the field remains in its early stages, and consensus has yet to be reached on numerous key scientific questions. This section focuses on five core controversies, revealing the underlying tensions in mechanistic understanding, strategic optimization, and clinical translation.

First, there is a debate between active chemotaxis and passive enrichment as the dominant tumor-targeting mechanism. The traditional view holds that bacteria actively sense and move toward the hypoxic tumor microenvironment through chemotaxis; however, experimental evidence challenges this notion: knockout of chemotaxis genes does not significantly reduce tumor colonization efficiency in *Escherichia coli* and *Salmonella*, whereas aromatic amino acid metabolic pathways and host macrophage status exert a more decisive influence. This suggests that bacterial targeting may rely primarily on passive enrichment resulting from vascular leakage. If chemotaxis is not critical for colonization, then the substantial body of genetic engineering strategies aimed at enhancing bacterial motility may warrant re-evaluation, and systematic comparisons across different bacterial strains and tumor models are urgently needed (16).

Second, cross-model discrepancies in colonization efficiency reflect deeper challenges in clinical translation. VNP20009 achieves high-density colonization of tumor in tumor-bearing mice, yet in phase I clinical trials, only a minority of patients had detectable bacteria in their tumors, as the rapid clearance of bacteria by the immunocompetent human body far exceeds that predicted by murine models. The blood–brain barrier, unique to gliomas, further exacerbates the uncertainty of colonization. This discrepancy warns that using colonization efficiency as a core endpoint for therapeutic evaluation may have fundamental limitations, and positive data from animal models cannot be directly extrapolated to clinical settings (102).

Third, attenuation strategies face a fundamental paradox between safety and efficacy. VNP20009 achieves attenuation by knocking out *msbB* and *purI*, yet this over-attenuation compromises its proliferative capacity, leading to colonization failure and lack of therapeutic efficacy in clinical trials. To overcome this bottleneck, researchers have on one hand constructed the ppGpp synthesis-deficient strain HCS1 to increase the tolerable dose window, and on the other hand explored non-genetic strategies such as macrophage camouflage to improve *in vivo* survival. However, whether high-dose bacterial colonization within the cranium can induce uncontrolled cerebral edema and neuroinflammation remains insufficiently assessed, and the optimal degree of attenuation remains elusive (22).

Fourth, the immunomodulatory direction presents a paradoxical picture of both activation and suppression. Taking *Listeria monocytogenes* as an example, intratumoral injection alone not only fails to suppress tumors but instead recruits Ly6G? neutrophils that polarize into PMN-MDSCs, accelerating tumor growth through inhibition of CD8? T cell function. However, if intravenous pre-immunization is performed beforehand, the induced systemic anti-*Listeria* T cells can clear these suppressive cells and convert bacterial colonization into a durable antitumor response. This implies that the immunological effect of oncolytic bacteria is not an intrinsic property, but rather a function of the host immune status, route of administration, and temporal sequencing. For the central nervous system, an immune-privileged organ, whether bacteria activate or suppress local immunity may largely depend on the patient’s individualized immune background, introducing substantial uncertainty for clinical translation (96).

Fifth, the role of bacterial toxins is debated between direct killing and immune adjuvancy. The traditional view attributes the oncolytic effect of pore-forming toxins to their direct disruption of tumor cell membranes; however, recent evidence suggests that the primary contribution of toxins may lie in triggering immunogenic cell death and releasing damage-associated molecular patterns, thereby acting as potent adjuvants that activate antitumor T cell responses. The clinical significance of this distinction lies in the following: if the core function of toxins is immune adjuvancy, then attenuated strains-even with diminished direct oncolytic capacity-may still exert therapeutic effects through immune pathways; conversely, if direct killing is indispensable, the trade-off between safety and efficacy becomes more acute. In the glioma microenvironment, toxin-triggered inflammatory responses may also precipitate cerebral edema, further complicating clinical decision-making.

In summary, the five controversies outlined above are not isolated-divergent views on the targeting mechanism influence attenuation strategies; the degree of attenuation determines whether bacteria can establish a population density sufficient to remodel the immune microenvironment; and the direction of immune activation or suppression depends on the dynamic interplay between bacteria and the host immune system. Resolving these controversies will require systematic mechanistic studies in orthotopic glioma models that more closely recapitulate the clinical setting, and ultimately, validation through well-designed clinical trials.

## 9 Summary and outlook

Oncolytic bacteria, with their inherent hypoxia-targeting ability, autonomous proliferation capacity, multiple antitumor mechanisms, and flexible genetic engineering modification potential, are highly compatible with the characteristic pathological microenvironment of malignant glioma. They effectively address the structural limitations of traditional therapies in terms of BBB penetration, tumor heterogeneity response, and immunosuppressive microenvironment remodeling. This article systematically reviews the core strain characteristics of oncolytic bacteria (differential colonization strategies of facultative and obligate anaerobes), antitumor molecular mechanisms (nutrient competition, toxin lysis, vascular embolism, ROS killing, immune remodeling, and trans-barrier delivery), and genetic engineering and synthetic biology modification strategies (strain attenuation, targeting enhancement, intelligent circuits, tandem viral delivery, and biomaterial composite modification). It summarizes the significant tumor-inhibitory effects in preclinical studies and the safety data from early clinical trials.

Despite the broad application prospects of oncolytic bacteria in glioma therapy, their clinical translation still faces multiple core challenges: the uncontrollable *in vivo* proliferation of live bacterial formulations and the risk of horizontal gene transfer constitute unique safety hazards; the residual barrier of the BBB and rapid clearance by the immune system result in intracranial colonization efficiency after intravenous administration far lower than predicted by animal models; bacterial-induced tumor necrosis and true progression are difficult to distinguish on imaging, and a standardized efficacy evaluation system is lacking; and oncolytic bacteria, possessing dual attributes as biological products and gene therapy products, lack a globally unified regulatory framework for production quality control, clinical operation, and informed consent norms.

Oncolytic bacterial therapy represents a distinctive therapeutic strategy for malignant glioma, with the potential to integrate tumor-selective colonization, direct tumor killing, reprogramming of the immunosuppressive microenvironment, and local synthesis of therapeutic agents. However, despite encouraging results in glioma animal models and early clinical experience with bacterial therapeutics in non-CNS malignancies, substantial obstacles remain for routine clinical translation. These include the blood–brain barrier, diffuse tumor infiltration, intratumoral heterogeneity, limited intracranial tolerance to inflammation, intrinsic or therapy-induced antibacterial immunity, and the need for rapid bacterial clearance in the event of toxicity.

Therefore, future development of oncolytic bacterial therapy for malignant glioma should move beyond merely enhancing bacterial replication capacity or intrinsic cytotoxicity, and instead evolve toward a controllable, multimodal, patient-adapted, and dynamically monitored therapeutic system. In the coming years, the following five research directions are particularly critical.

### 9.1 Engineering bacteria for *in situ* synthesis of immunomodulatory molecules

One major future direction is the construction of tumor-targeting bacteria that serve as “living factories” for local synthesis of immunomodulatory molecules. The immunosuppressive microenvironment of glioma is characterized by abundant tumor-associated macrophages and microglia, impaired antigen presentation, T cell exhaustion, insufficient effector cell infiltration, and upregulation of inhibitory immune pathways. Systemic administration of cytokines or immune checkpoint inhibitors is often limited by inadequate intracranial drug delivery, accompanied by dose-dependent systemic toxicity. In contrast, bacteria that preferentially accumulate in the tumor microenvironment can serve as living delivery vehicles for the spatiotemporally controlled release of cytokines, chemokines, co-stimulatory ligands, antibody fragments, or immune checkpoint inhibitors.

Therapeutic payloads that bacteria can carry include IL-12, GM-CSF, interferon-related molecules, T cell-recruiting chemokines, and nanobodies or single-chain antibodies targeting inhibitory checkpoints such as PD-1, PD-L1, and CTLA-4. Local synthesis of these molecules within the tumor can promote dendritic cell activation, antigen presentation, macrophage repolarization, and cytotoxic T cell recruitment, converting immunologically “cold” gliomas into more inflamed lesions. Programmable bacteria expressing immunomodulatory molecules have induced durable antitumor responses in various tumor animal models, confirming that bacterial colonization can be coupled with local checkpoint inhibition and systemic antitumor immunity (115). In glioma models, engineered bacteria combined with PD-1 blockade similarly promote M1 macrophage polarization and enhance antitumor T cell responses (116).

However, immune activation in the brain must be precisely regulated. Excessive cytokine release can induce neuroinflammation, vasogenic edema, seizures, and even neurological deterioration; conversely, insufficient payload expression fails to overcome local immunosuppression. Therefore, next-generation strains should integrate hypoxia-, acidity-, lactate-, or necrosis-responsive promoters, coupled with quorum-sensing circuits or inducible lysis systems, to restrict therapeutic payload release to the tumor site and avoid uncontrolled bacterial proliferation (78, 117). In this context, the most clinically valuable bacterial carriers are not those with the strongest replication capacity, but rather those capable of generating immunostimulatory signals of appropriate intensity, reversibility, and spatial confinement.

### 9.2 Combination of oncolytic bacteria with standard-of-care therapies and tumor treating fields

A second highly promising direction is the mechanism-driven combination of bacterial therapy with radiotherapy, chemotherapy, and Tumor Treating Fields (TTFields). Malignant glioma exhibits spatial and biological heterogeneity, and bacterial monotherapy is unlikely to eliminate all tumor subpopulations. Anaerobic strains preferentially colonize hypoxic necrotic regions, whereas infiltrating tumor cells at the tumor margin reside in a relatively more oxygenated microenvironment that bacteria find difficult to reach. Combination strategies are therefore required to target distinct tumor microenvironment niches and circumvent therapeutic escape.

Radiotherapy is highly compatible with oncolytic bacterial combination. Radiotherapy can induce tumor cell stress, immunogenic cell death, vascular remodeling, and tumor antigen release, potentially enhancing bacterial infiltration efficiency and promoting immune recognition of residual glioma cells. Conversely, bacterial therapy can reshape the hypoxic and immunosuppressive microenvironment prior to radiotherapy, rendering residual tumor cells more sensitive to radiation damage. Future studies need to determine whether bacterial therapy should be administered before radiotherapy as an immune pre-conditioning step, or after radiotherapy to leverage radiation-induced necrosis and inflammatory signals.

Chemotherapy combination regimens likewise require systematic investigation. Engineered bacteria expressing prodrug-converting enzymes, cytotoxic proteins, or drug-sensitizing molecules can achieve *in situ* activation of chemotherapeutic agents while reducing systemic exposure. This strategy is particularly relevant for temozolomide-resistant gliomas: bacterial modulation of DNA damage responses, metabolic pathways, or antitumor immunity can complement conventional alkylating agent therapy. However, chemotherapy can also impair immune cell function, accelerate bacterial clearance, and potentially increase infection risk. Therefore, treatment schedules, doses, and sequencing must be optimized experimentally rather than selected empirically.

Tumor Treating Fields (TTFields) represent another highly promising combination partner. TTFields are non-invasive alternating electric fields that interfere with cell mitosis and have demonstrated clinical benefit in newly diagnosed glioblastoma when combined with maintenance temozolomide (118). The mechanism of action of TTFields is distinct from bacterial colonization and immunomodulation, and their combination is expected to produce additive or synergistic effects without necessarily increasing systemic toxicity. Furthermore, TTFields can modulate DNA damage responses, cellular stress pathways, and the tumor immune microenvironment, providing a basis for mechanism-driven combination regimens. However, no direct evidence for bacterial–TTFields combination in glioma currently exists. Therefore, this approach should first be evaluated in immunocompetent orthotopic glioma models, with emphasis on bacterial distribution, immune cell composition, cerebral edema, seizure risk, and treatment-related neurotoxicity.

Overall, future combination studies should move beyond simple drug stacking toward mechanism-driven sequential treatment regimens. A clinically feasible strategy might involve: postoperative local bacterial administration to induce tumor inflammation and antigen release; followed by radiotherapy or temozolomide to eliminate residual tumor cells; and finally TTFields or systemic immunotherapy as maintenance therapy. The core question is how to maximize durable antitumor immunity without compromising T cell function or inducing excessive intracranial inflammation.

### 9.3 Development of non-invasive and minimally invasive delivery strategies

A third key direction is the construction of delivery platforms that enhance intracranial bacterial exposure while reducing systemic bacterial dissemination and neurological damage risk. Intravenous injection is simple to perform and theoretically capable of addressing multifocal lesions; however, it is hindered by bacterial sequestration in the liver and spleen, immune-mediated bacterial clearance, inefficient blood–brain barrier crossing, and the risk of systemic inflammatory toxicity. Therefore, for malignant glioma, local and regional delivery strategies represent a more controllable starting point for clinical translation.

Convection-enhanced delivery (CED) holds significant promise: pressure-driven direct infusion of drugs into tumor tissue or peritumoral infiltrated brain tissue can bypass the blood–brain barrier and achieve drug distribution beyond the injection site. Clinical trials in recurrent glioblastoma have employed prolonged CED for local administration, confirming that catheter-based, image-guided intracranial infusion can be adapted for bacterial or bacteria-derived therapeutic systems (119). For bacterial therapy, CED can deliver attenuated bacteria, bacterial spores, bacterial extracellular vesicles, or bacteria-loaded biomaterials to the tumor bed, surgical resection cavity, or tumor-infiltrated margins. However, the impact of convective infusion on bacterial viability, distribution, proliferation, and local inflammatory responses requires dedicated evaluation.

Postoperative administration via the surgical resection cavity represents another practical strategy. Injectable hydrogels, biodegradable scaffolds, or sustained-release formulations can retain bacteria or bacteria-derived products within the resection cavity and continuously release them to residual lesions at the surgical margins. A preclinical study in glioblastoma integrated a local bacterial therapeutic platform into an ATP-responsive hydrogel, which activated tumoricidal immunity and inhibited postoperative tumor recurrence, demonstrating the application potential of local postoperative delivery (18).

Intranasal administration may serve as a complementary non-invasive route for bacteria-derived therapeutics, particularly for non-replicative bacterial vesicles, membrane-coated nanoparticles, or engineered microbial products. The nasal-to-brain transport pathway can circumvent some limitations of systemic administration. Although intranasal administration of live oncolytic bacteria has not been fully validated in glioma, intranasal nanoparticle delivery has achieved tumor accumulation and therapeutic efficacy in orthotopic glioblastoma models (120). Therefore, intranasal bacterial therapy remains an exploratory direction rather than a mature translational strategy. Its future value depends on achieving stable brain delivery, avoiding nasal and systemic toxicity, and ensuring effective therapeutic concentrations in both the tumor core and infiltrating margins.

### 9.4 Integration of imaging and biosensing functions to establish theranostic bacterial therapeutic platforms

A fourth key direction is the development of theranostic bacterial platforms that combine therapeutic and real-time monitoring capabilities. A major challenge in bacterial tumor therapy is the inability to determine in real time whether bacteria have successfully reached the tumor, whether they maintain effective population density, whether they release therapeutic payloads, and whether they have disseminated to non-target organs. This problem is particularly acute in the brain, where uncontrolled bacterial proliferation or excessive inflammation can cause severe neurological damage.

Engineered bacteria can be equipped with reporter systems that produce fluorescent, bioluminescent, magnetic, or radionuclide-adaptable signals for non-invasive tracking of bacterial localization, tumor colonization, therapeutic payload expression, and bacterial clearance. Studies have demonstrated that engineered *Salmonella* can simultaneously deliver cytotoxic genes and reporter genes as imageable therapeutic probes (46). Tumor-responsive bacterial promoters can also generate reversible reporter signals in response to tumor microenvironment signals such as acidity, providing a framework for conditional biosensing and therapy (121).

For malignant glioma, future theranostic platforms could integrate bacteria-specific imaging with conventional magnetic resonance imaging, PET, and intraoperative fluorescence imaging. Additionally, bacterial DNA, RNA, proteins, metabolites, and circulating immune markers can be detected in blood; cerebrospinal fluid may also be analyzed when clinically feasible. These biomarkers can serve as pharmacodynamic indicators reflecting bacterial load, payload expression, inflammatory activation, and therapeutic response.

The long-term goal is to construct a closed-loop therapeutic system: imaging or biosensing confirms bacterial tumor localization; exogenous inducers, antibiotics, or kill switches are then used to modulate bacterial activity; repeat imaging verifies bacterial clearance or guides re-administration. This strategy enables dynamic adjustment of treatment intensity based on bacterial persistence, tumor response, and adverse inflammatory events, simultaneously enhancing safety and efficacy.

### 9.5 Personalized bacterial therapy based on tumor microbiome and immune characteristics

A fifth future direction is personalized bacterial therapy based on the patient’s tumor immune status, molecular features, and microbiome profiles. Glioblastoma patients exhibit significant inter- and intratumoral heterogeneity across multiple dimensions, including necrosis, hypoxia, vascular permeability, myeloid cell infiltration, T cell exhaustion, antigen presentation, and metabolic activity. These factors directly determine whether a given bacterial carrier can successfully colonize the tumor, survive in the local microenvironment, induce effective immune activation, or produce unacceptable toxicity.

Therefore, patient screening for early bacterial therapy clinical trials should incorporate imaging and molecular biomarkers. Relevant parameters include the extent of necrosis and hypoxia, perfusion and vascular permeability, immune cell composition, mesenchymal/inflammatory transcriptional programs, glucocorticoid exposure, target antigen expression, and innate antibacterial immunity. Tumors with extensive hypoxia and necrosis are more suitable for anaerobic bacterial spores; tumors with low necrosis and high invasiveness are better suited for facultative anaerobes, bacterial vesicles, or bacteria-derived nanomaterials. Similarly, immunologically cold tumors are appropriate for engineered bacteria expressing cytokines or checkpoint inhibitors; tumors with pre-existing immune infiltration may be better combined with radiotherapy, immune checkpoint blockade, or TTFields.

The tumor-associated microbiome and gut microbiome can further support personalized therapy. Bacterial-derived peptides have been detected on HLA molecules in glioblastoma tissue and are recognized by tumor-infiltrating lymphocytes, indicating that microbial antigens participate in glioma immune recognition (122). Clinical data also suggest that immune, genomic, and gut microbiome features correlate with prognosis in glioblastoma patients receiving PD-L1 inhibitors combined with chemoradiotherapy (123). Although these data do not yet establish a causal relationship between specific microbiome features and bacterial therapy response, they support prospective microbiome profiling in future clinical trials.

Ultimately, personalized bacterial therapy requires matching bacterial species, genetic circuits, therapeutic payloads, administration routes, and combination treatment sequencing to the patient’s tumor immune and metabolic phenotype. Bacterial colonization cannot be simplistically regarded as a uniform biological effect; future studies need to identify which patient subgroups are most likely to achieve effective bacterial colonization, local payload release, and clinically meaningful immune activation.

In summary, oncolytic bacterial therapy for malignant glioma is evolving from a concept focused on bacterial tumor colonization toward a more comprehensive therapeutic system encompassing local immune engineering, multimodal combination therapy, delivery technology optimization, real-time monitoring, and personalized treatment design. The most realistic near-term goal is not to replace surgery, radiotherapy, temozolomide, or TTFields, but to develop bacterial platforms as complementary tools: targeting the hypoxic microenvironment, remodeling local immunosuppression, and achieving sustained intratumoral drug release. The next phase of clinical translation should prioritize safe and controllable bacterial carriers, GBM-specific regional delivery systems, biomarker-based patient enrollment, and adaptive monitoring strategies. If these challenges are overcome, engineered bacteria and bacteria-derived therapeutics are poised to become important components of precision combination therapy for postoperative residual disease and recurrent malignant glioma.
